# A Rare Case of ARDS Caused by Bupropion Inhalation and Treated with Noninvasive Ventilation

**DOI:** 10.1155/2020/5107456

**Published:** 2020-05-28

**Authors:** Yousif Al-Saiegh, Jenna Spears, Pieter S. De Klerk, Joshua Hitchings, Christopher Lee, Tamara Mahr

**Affiliations:** ^1^Department of Medicine, Pennsylvania Hospital, University of Pennsylvania Health System (UPHS), Philadelphia PA, USA; ^2^Department of Pulmonary/Critical Care, Pennsylvania Hospital, University of Pennsylvania Health System (UPHS), Philadelphia PA, USA

## Abstract

Acute respiratory distress syndrome, characterized by the Berlin criteria, is associated with a high mortality rate. Its treatment includes addressing the underlying etiology, general supportive measures, and achievement of effective oxygenation. New key data indicates that in a subset of patients, noninvasive ventilation techniques can be a therapeutic and equivalent alternative to traditional invasive ventilation. We present a rare case of ARDS triggered by nasal bupropion inhalation and effectively treated with noninvasive positive pressure ventilation resulting in complete resolution.

## 1. Introduction

Acute respiratory distress syndrome (ARDS) incorporates a cluster of clinical features including shortness of breath and tachypnea and is defined by the Berlin criteria as having an acute onset with the development of hypoxemia and bilateral pulmonary opacities on radiographic imaging [1]. There are many causes of ARDS; however, most often, it is triggered by infections, blood transfusions, direct lung injury, and toxins [2]. Treatment includes removal of the inciting cause, supportive therapy, and the attainment of sufficient blood oxygenation. Adequate oxygenation in ARDS is often achieved by endotracheal intubation and mechanical ventilation. Noninvasive positive pressure ventilation (NIPPV) has been less frequently indicated as an alternative form of adequate oxygenation. Typically, its use is focused and intended on preventing complications that are associated with invasive ventilation such as barotrauma, vocal cord injury, and ventilator-associated pneumonia [3].

Bupropion is an atypical oral antidepressant medication commonly used to treat depression, tobacco dependence, obesity, and hypoactive sexual disorder. Its mechanism of action involves the inhibition and reuptake of norepinephrine and dopamine which can induce a state of euphoria. Because of these effects, bupropion has been reported to be abused recreationally [4, 5]. We describe a case of ARDS induced by bupropion inhalation that was treated with NIPPV.

## 2. Case Presentation

A 30-year-old male with a past medical history significant for polysubstance abuse presented to the emergency department (ED) two hours after ingesting 90 mg of oxycodone and 30 mg of diazepam along with intranasal bupropion. On arrival, he complained of extreme fatigue, respiratory distress, and confusion.

**Figure 1:**
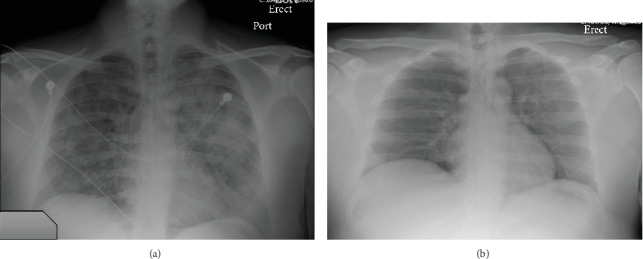
(a) Single-view anterior-posterior chest X-ray on day #1 showed diffuse bilateral lung opacities. (b) Repeat single-view anterior-posterior chest X-ray on day #6 showed decreased airspace opacities.

**Figure 2:**
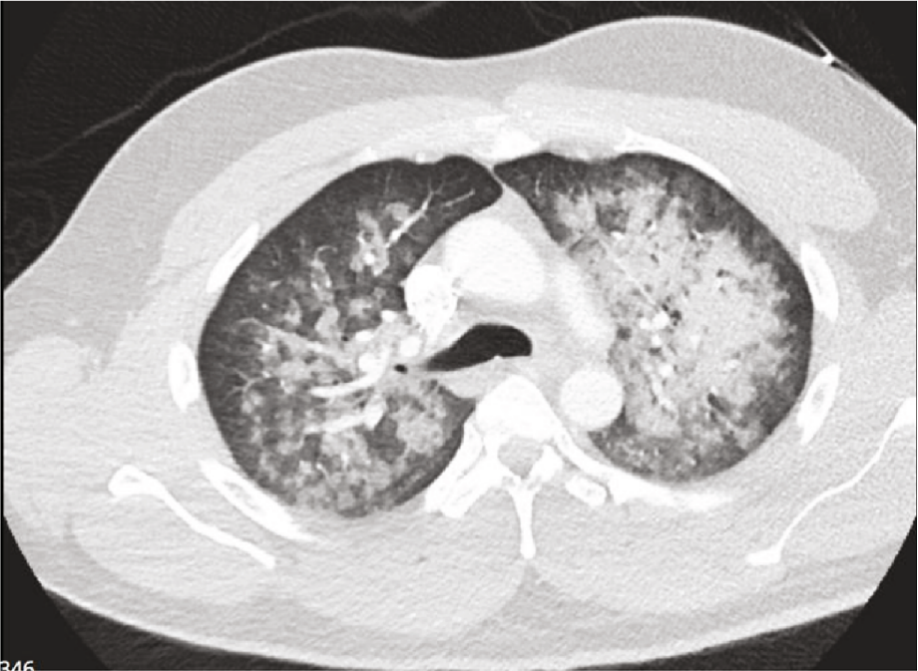
Contrast-enhanced computed tomography of the chest on day #2 shows diffuse bilateral airspace disease characterized by groundglass and consolidative opacities, with relative peripheral sparing and perihilar predominance. No pleural effusions or pneumothorax.

In the ED, he was noted to be lethargic and short of breath. Initial vital signs revealed heart rate of 104 beats per minute, respiratory rate of 35 per minute, and O_2_ saturation of 75% on room air. Respiratory exam revealed diffuse bilateral coarse breath sounds on inspiration and expiration. His laboratory results were notable for a leukocytosis of 14.1 K, creatinine of 1.25 g/dl, lactate of 4.1, and an elevated procalcitonin level. Venous blood gas analysis showed a pH of 7.18 with a pCO_2_ of 51 mmHg. Chest X-ray (CXR) demonstrated diffuse bilateral alveolar infiltrates (Figure 1(a)). Computed tomography (CT) of the chest showed diffuse bilateral airspace disease characterized by groundglass and consolidative opacities with relative peripheral lung sparing and perihilar predominance (Figure 2). He received naloxone with mild improvement in mental status and was initiated on Bilevel Positive Airway Pressure (BiPAP) with an inspiratory positive airway pressure (IPAP) of 15 cm H_2_O, expiratory positive airway pressure (EPAP) of 5 cm H_2_O with 40% fraction of inspired oxygen (FiO_2_). Empiric broad spectrum antibiotics including vancomycin, cefepime, and metronidazole were initiated along with intravenous methylprednisolone 40 mg every 12 hours.

Initially while on BIPAP, he remained tachypneic and tachycardic. His respiratory rate improved over the following 6 hours. He was gradually weaned off BiPAP to nasal cannula oxygen over the course of 36 hours while receiving ongoing corticosteroid and antibiotic therapy.

Repeat CXR on hospital day #6 showed markedly improved bilateral airspace opacities (Figure 1(b)). After 6 days, he was discharged in stable condition without requiring supplemental oxygen.

## 3. Discussion

ARDS is associated with a high mortality that has declined from over 50% to 30% over the last four decades [6, 7]. This is primarily due to implementation of lung-protective ventilation protocols and intensified research after formation of the National Heart, Lung, and Blood Institute (NHLBI) ARDS network [8, 9]. This case highlights a mild manifestation of ARDS as a result of bupropion inhalation, an exceedingly rare etiology.

The Food and Drug Administration (FDA) classifies bupropion as a psychiatric medication with low abuse potential [10]. However, several case reports and studies have indicated increasing recreational use of bupropion mostly intravenously or intranasally [11–13]. Lewis et al. conducted a review on bupropion inhalation in a total of 67 patients. Seizures were noted as a common adverse effect occurring in 30% of patients. Acute lung toxicity was not reported as a complication [14]. We were not able to find a second reported case of bupropion-inhalation-induced ARDS.

Endotracheal intubation and mechanical ventilation are mostly required to ensure adequate oxygenation, but this was avoided in this patient as we maintained adequate oxygenation with BiPAP alone. NIPPV is advantageous in such patients as it does not expose them to the potential complications of invasive ventilation and may shorten their hospital length of stay [3]. There is, however, an ongoing debate concerning the most effective mode of NIPPV [15, 16]. Recent studies show that noninvasive ventilation can be used in mild cases of ARDS with acute nonhypercapnic hypoxemic respiratory failure [17]. In these cases, BiPAP via facemask is the most commonly used strategy [18]. Another approach with high-flow nasal cannula was shown to have a similar degree of treatment failure and incidence of subsequent intubation as BiPAP. High-flow nasal cannula, however, was associated with decreased 90-day mortality as compared to BiPAP [19]. A randomized, single-center trial by Pohlman et al. showed that delivering noninvasive ventilation via helmet instead of by facemask was associated with a significant reduction in intubations. There was also a decrease in intensive care unit length of stay and mortality at 90 days [20]. However, a subset analysis of the observational “LUNG-SAFE” study for patients with severe ARDS (defined as a PaO_2_/FiO_2_ ratio below 150 mmHg) showed increased mortality with noninvasive ventilation [21].

Per our review, data on the use of noninvasive ventilation in the management of ARDS remains inconclusive and conflicting. NIPPV may decrease the incidence of ventilator-associated complications; however, given that its efficacy is not well established, patients with ARDS should still be treated in a critical care setting for close monitoring with invasive ventilation available on standby.

## 4. Conclusion

Advances in the treatment of the underlying etiologies and improvement in mechanical ventilation strategies have led to a decrease in the overall mortality associated with ARDS. Despite these developments, it remains a diagnosis with an exceedingly high mortality. New emerging data indicates that NIPPV can be a beneficial and equivalent approach for a subset of patients with ARDS, although the optimal type of noninvasive ventilation and patient group that would benefit most is yet to be determined.
